# Are Dryland Strength and Power Measurements Associated with Swimming Performance? Preliminary Results on Elite Paralympic Swimmers

**DOI:** 10.3390/sports12040094

**Published:** 2024-03-26

**Authors:** Luca Cavaggioni, Raffaele Scurati, Massimiliano Tosin, Riccardo Vernole, Luca Bonfanti, Athos Trecroci, Damiano Formenti

**Affiliations:** 1 Obesity Unit—Laboratory of Nutrition and Obesity Research, Department of Endocrine and Metabolic Diseases, IRCCS Istituto Auxologico Italiano, 20145 Milan, Italy; 2 Department of Biotechnology and Life Sciences, University of Insubria, 21100 Varese, Italy; luca.bonfanti@uninsubria.it (L.B.); damiano.formenti@uninsubria.it (D.F.); 3 Department of Biomedical Sciences for Health, Università degli Studi di Milano, 20133 Milan, Italy; raffaele.scurati@unimi.it (R.S.); athos.trecroci@unimi.it (A.T.); 4 Italian Paralympic Swimming Federation, 00144 Rome, Italy; lombardia@finp.it (M.T.); riccardo.vernole@finp.it (R.V.)

**Keywords:** Paralympic swimming, disability sport, bench press, lat pull-down, dryland training

## Abstract

This study aimed to identify the relationship between dryland tests and swimming performance in elite Paralympic swimmers. Fifteen competitive swimmers (age: 27.4 ± 5.4 years, height: 1.70 ± 6.8 m, body mass: 67.9 ± 9.2 kg; 9 males, 6 females) performed a lat pull-down and a bench press incremental load test to determine maximum power (Pmax), the strength corresponding to maximum power (F@Pmax), and the barbell velocity corresponding to maximum power (V@Pmax) from the force–velocity and power–velocity profiles. These outcomes were also normalized by the athlete’s body mass. Swimming performance was carried out from the best result in a 100 m freestyle race registered during an international competition. Lat pull-down F@Pmax was significantly associated with 100 m freestyle chronometric time (*ρ* = −0.56, *p* < 0.05), and lat pull-down V@Pmax presented a relationship with mean swimming velocity (*ρ* = 0.71, *p* < 0.01). Similarly, bench press F@Pmax and the normalized F@Pmax were significantly related to the mean swimming velocity (*ρ* = −0.51, *ρ* = −0.62, *p* < 0.05). Stepwise multiple regression showed that lat pull-down V@Pmax, bench press normF@Pmax, and V@Pmax accounted for 40.6%, 42.3%, and 65.8% (*p* < 0.05) of the mean swimming velocity variance. These preliminary results highlighted that simple dryland tests, although with a moderate relationship, are significantly associated with 100 m freestyle swimming performance in elite Paralympic swimmers.

## 1. Introduction

The number of Para-athletes competing in the quadrennial Paralympic Games, an event for athletes with disabilities, has increased significantly since 1960 [1]. Moreover, swimming is one of the oldest sports and was the event with the greatest number of competitors at Rio 2016 (more than 500 athletes), second only to athletics [2].

Swimming performance is influenced by a number of elements, including physiological, neuromuscular, technical, and psychological ones [3,4,5]. Among the neuromuscular factors, elite swimmers are required to express high levels of muscular power and strength to display an adequate and efficient swimming performance [6,7,8]. From this point of view, the upper body segments are particularly determinant in overcoming propulsive forces [4]. In competitive swimming, there are different approaches to improving muscle strength: in-water solutions and muscular training conducted on dryland [9].

In both able-body and Para swimming, many lines of research have highlighted the importance of developing muscular strength for performance enhancement [10,11,12,13,14,15]. In this context, Para swimmers with severe impairments are helped to ameliorate their swimming propulsion thanks to increased muscular power, especially during the swim start [11,16]. The nature and the level of physical impairment (i.e., high and low range) may compromise the force production and swimming velocity, displaying more asymmetries [10]. In light of this, intervention studies on Para swimmers have demonstrated the effectiveness of dryland training for improving upper and lower body muscular strength, ranging from a shorter (e.g., 6 weeks) to a mid to long time period (e.g., from a few months to an entire season) [13,14,15,17]. Daly et al. (2001) highlighted the importance of muscle power in Paralympic swimmers in ameliorating some technical aspects, especially during turning and finishing or maintaining swimming speed [18]. Research has shown that muscular weakness in adolescence can be linked to disability later in life, underscoring the long-term consequences of strength levels [19]. Furthermore, studies emphasize the importance of developing muscle mass and upper body power to improve Para-athletes’ swimming performance [14,15,17]. In addition, outside the competition realm, muscular strength not only enhances performance but also plays a vital role in the quality of life for individuals with disabilities [20].

These neuromuscular features (e.g., strength and power) may be significantly harmed by physical and sensory disabilities such as cerebral palsy, multiple sclerosis, spinal cord injury, ataxia, traumatic brain injury, and visual impairment. According to a study by Portilla-Cueto et al. (2022), multiple sclerosis frequently impairs muscle function, particularly in the lower limbs, which results in a decline in muscle quality and strength [21]. Bergwell et al. (2022) showed that cerebral palsy patients had poor somatosensory cortex activity, which is linked to a decrease in maximum strength [22]. García et al. (2019) also pointed out that a spinal cord injury had a significant negative effect on muscle function [23]. Additionally, individuals with ataxia may also develop locomotor abnormalities that impair both neurological and muscular function, which may have a negative impact on overall muscular performance [24]. Lastly, a person with a visual impairment may struggle with his muscle strength and power [25]. Furthermore, Horvat et al. (2006) observed lower body strength and power in individuals with visual impairments, suggesting that these factors may contribute to motor control difficulties experienced by this population [26]. These conditions not only compromise muscular strength and power but also hinder overall motor function, underscoring the importance of targeted interventions to address these challenges and improve motor performance.

Although the importance of developing strength and power for swimming performance is well known, concerning able-body swimming, a growing body of research highlights conflictual results in the association between dryland strength measurements and swimming performance [6,8,27,28,29,30,31]. On one hand, Garrido et al. found a significant moderate correlation between muscular tests detecting strength and power outcomes on dryland and 25 m or 50 m freestyle sprint tests in competitive swimmers [28]. Similarly, Pérez-Olea and colleagues showed significant relationships between mean velocity and power in pull-ups with 50 m swimming performance [32], and Morouço et al. showed significant associations between in-water and dryland tests in lat pull-down mean power and swimming velocity in a 50 m freestyle test in national-level swimmers [22]. Additionally, Loturco and colleagues investigated the correlations between dryland tests and specific swimming performance, highlighting the importance of strength–power exercises in training routines [33]. Furthermore, a more recent study demonstrated strong relationships between force variables in dryland exercises (like bench presses and squat jumps) and force production during tethered swimming [34]. These findings emphasize the significance of dryland training and its impact on swimming performance, indicating that improvements in dryland variables can lead to enhanced swimming outcomes. The relationship between dryland variables and swimming performance underscores the importance of incorporating strength and power training on land to optimize athletic performance in the water.

On the other hand, other cumulating evidence did not reveal associations between dryland exercise outcomes and swimming performance, reporting weak-to-nonsignificant correlations [4,19,22,23,24]. Presumably, this inconclusive evidence on the association between in-water performance and dryland may be related to their different nature. On-land strength is not always directly related to swimming performance due to different exercises’ biomechanics and technical aspects, athlete’s competition level, or anthropometric features, which limit the transferal process between on-land issues into aquatic performance [9,35].

Nevertheless, taking into account that swimming performance is composed of multi-factorial elements [3], the possibility of investigating the relationship between each component (e.g., technical, physical, biomechanical, psychological, and nutritional) could be beneficial for coaches to optimize athlete’s performance by reducing training complexity, especially in Para swimming, where, beyond the swimming technique, there is also the impairment’s nature that could affect the final result.

To the best of our knowledge, no study has examined the relationship of on-land strength and power with swimming performance in Para swimmers. As for the literature on able-body swimmers [27,28,29,30], the present study intends to extend knowledge on the relationship between dryland muscular performance and swimming performance. Given the positive impact of dryland training exercises on overall swimming performance, this research could be of interest to sports scientists and practitioners on the potential importance of evaluating and monitoring dryland muscular performance for Para swimming athletes.

Therefore, the present study aimed to identify whether an association exists between strength and power in dryland upper body exercises and swimming performance in elite Paralympic swimmers.

## 2. Materials and Methods

### 2.1. Participants

The study involved 15 international-level elite Paralympic swimmers (age: 27.4 ± 5.4 years; height: 1.70 ± 6.8 m; body weight: 67.9 ± 9.2 kg; 9 males, 6 females). Each athlete participated at a major international (e.g., European Championship, World Championship, or Paralympic Games) or national event (e.g., Italian Championships) at least once. They had at least three years of background in competitive swimming, reaching the podium in one of the above-mentioned competitions at least once in the four-year Paralympics cycle. In Para swimming, there are ten functional classes named with the prefix “S” (freestyle, backstroke, and butterfly) or SB (breaststroke) followed by a number that ranges from 10 to 1 based on the swimmer’s degree of functional disability. The lower the class number (e.g., S2), the greater the limitation caused by the athlete’s physical impairment. In addition, the S11–S13 classes are dedicated to visually impaired athletes, and the S14 class belongs to intellectual impairments. The athletes recruited in our study were as follows: one in the S2 class; one in the S3 class; two in the S4 class; two in the S5 class; one in the S6 class; four in the S7 class; one in the S8 class; two in the S9 class; and one in the S13 class. The exclusion criteria were the presence of recent injury in the upper limbs on the day of testing or any other clinical condition that would compromise the practice testing. Written informed consent was provided, and all the assessment procedures were in accordance with the Declaration of Helsinki. The ethics committee of the local university approved the investigation.

### 2.2. Design-Setting and Dryland Procedures

This study involved testing procedures performed in a single experimental session conducted in the same indoor gym with similar conditions (temperature 21–25 °C, relative humidity 41–50%) and at the same time of day (3.00 p.m. to 5.00 p.m.). All the athletes were requested to abstain from consuming alcoholic or caffeinated drinks before the testing day and to continue following a balanced mixed diet consisting of a variety of nutrients in terms of carbohydrate intake and high-biological-value protein that was sufficient to meet the micro and macronutrients required. To avoid any potential fatigue effects, the athletes were also requested to not engage in any type of exhaustive physical activity, exercise training, or sports competition in the 24 h prior to the testing procedures.

The dryland assessment comprised two multi-articular upper body exercises: the lat pull-down and bench press with barbell. These exercises were chosen because they have been performed in previous research showing moderate-to-high adherence to aquatic performance in able-bodied swimmers [28,30,34].

The participants followed a standardized warm-up of 10 min, planned as follows: active joint mobility (e.g., shoulder girdle, cervical and thoracic spine), followed by two sets from ten to fifteen repetitions of lat pull-down and bench press exercises (training intensity ~50% of 1 maximum repetition, 1RM), observing three minutes of rest in between. According to a previous study on Para swimmers [13], each athlete performed three repetitions at maximal speed for each incremental load, starting from a criterion load of 30% of his/her body mass. The load was progressively augmented by 5% of the previous load in each set until a decrease of 0.5 m/s in mean propulsive velocity was reached. In detail, during the lat pull-down exercise, participants lowered the bar with a continuous motion in a controlled manner until touching the chest, and after the command “start”, they were required to move the bar as fast as possible. Specifically, feedback based on concentric velocity was provided. This was accomplished by using a wearable inertial sensor (described below in detail) that registered the kinematics of every repetition, and its software provided visual and auditory feedback in real time. The eccentric phase was performed at a controlled velocity, but it was requested to execute the concentric phase of each repetition at the maximal intended velocity, in a rapid manner. In addition, strong verbal encouragement and feedback were provided in each repetition in an attempt to motivate the participants to produce their maximal effort. As previously mentioned, each swimmer performed three repetitions with each selected load. Only the best repetition at each load, according to the criteria of fastest mean propulsive velocity, was further considered for the analysis [36]. Five minutes of rest were allowed in between to achieve a complete recovery. Technically experienced coaching staff was present for assistance in case of technical failure to limit the risk of injury. In addition, body posture safety and position were guaranteed during each repetition for both exercises. Specifically, during the lat pull-down, all the athletes with limited to no lower limb function adopted leg straps to ensure that their legs remained in a proper position. Simultaneously, a qualified adaptive coaching staff properly supervised and spotted the athletes during the whole testing protocol. After a 20-min rest composed of active recovery, joint mobility, stretching, and core-stability exercises, each swimmer replicated the same testing methodology of lat pull-downs in the bench press exercise until reaching a mean propulsive velocity reduction lower than 0.6 m/s. Regarding this exercise, thorax and leg straps were used to guarantee lower body contact with the bench, while experienced personnel with adaptive athletes ensured all the safety procedures.

A wearable wireless sensor (Gyko system, Microgate, Bolzano, Italy), for which validity and reliability were previously established [37,38,39], was attached to the barbell using a built-in magnet. The Gyko system is an inertial sensor composed of a three-dimensional accelerometer, gyroscope, and magnetometer, which allows recordings (full-scale range: 8 g) at a sampling frequency of 500 Hz. During the testing procedures, the accelerometer and gyroscope signals were transferred via Bluetooth to a personal computer (Toshiba model) and stored using the appropriate software (GykoRePower Software 1.2.2.0). The software automatically calculated the mean propulsive velocity, power, and force.

Finally, from the individual force–velocity and power–velocity profiles, the maximum power (Pmax), the force that corresponds to maximum power exertion (F@Pmax), and the barbell velocity displacement linked to maximum power (V@Pmax) were calculated [40]. A normalization process using body weight was also performed from the above-mentioned parameters, calculating normPmax and normF@Pmax (dividing Pmax and F@Pmax by body mass).

### 2.3. Swimming Performance Evaluation

Swimming performance was determined from official results in a 100 m freestyle race in the Olympic pool (50 m) or by recording the best performance within the past two years during an international event (i.e., 2022 or 2023 World Championships or during a World Series event in the same time period). Official swimming chronometric times have been extrapolated from the websites https://www.finp.it (accessed on 3 January 2024) and https://www.natatoria.org (accessed on 4 December 2023), and mean swimming velocity was estimated using the formula v100 = 100.∆t^−1^, where ∆t is the chronometric time [30].

### 2.4. Statistical Analysis

The data are presented as the mean ± standard deviation, and the normality of the distribution was checked using the Shapiro–Wilk test. Spearman correlation coefficients (*p*) were calculated between in-water and dryland outcomes. Moreover, a stepwise multiple regression was used to predict 100 m freestyle swimming performance. The independent variables that correlated most significantly with the 100 m freestyle chronometric time and the 100 m mean velocity were entered into the stepwise calculation. Correlation coefficients of 0.1, 0.3, 0.5, 0.7, and 0.9 were considered small, moderate, large, very large, and extremely large [41]. All statistical analyses were performed using the Statistical Package for Social Sciences, IBM SPSS Statistics (version 21.0, IBM Corp., Somers, Chicago, IL, USA).

## 3. Results

The mean 100 m freestyle chronometric time was 90.6 ± 52.4 s, and the mean swimming velocity was 1.31 ± 0.4 m/s. As for dryland exercises, in the lat pull-down, the maximum power (Pmax), the force corresponding to maximum power exertion (F@Pmax), the barbell velocity displacement corresponding to maximum power (V@Pmax), the normalized maximum power (normPmax), and the normalized F@P (normF@Pmax) were 1284 ± 1303 W, 828.6 ± 416 N, 0.98 ± 0.2 m/s, 18.2 ± 16.5 W/Kg, and 12.2 ± 5.5 N/Kg. For the barbell bench press, the mean Pmax was 434 ± 169 W, the F@Pmax was 528 ± 166 N, the V@Pmax was 0.74 ± 0.1 m/s, the normPmax was 6.4 ± 2.3 W/Kg, and the normF@Pmax was 7.3 ± 2.8 N/Kg.

Table 1 presents the correlation coefficients (*p*) between the swimming performance and dryland variables (Pmax, F@Pmax, V@Pmax, normPmax, normF@Pmax) in both exercises. Specifically, a significant (*p* < 0.05) large association was found between the 100 m chronometric time and lat pull-down normF@Pmax (*ρ* = −0.567), a significant (*p* < 0.01) very large relationship between mean swimming velocity and lat pull-down V@Pmax (*ρ* = 0.712), a large significant (*p* < 0.05) correlation between mean swimming velocity and bench press F@Pmax (*ρ* = −0.518), and a significant (*p* < 0.05) large relationship between mean swimming velocity and bench press normF@Pmax (*ρ* = −0.623).

Lastly, the stepwise multiple regression model with the 100 m mean swimming velocity as a dependent variable highlighted that the V@Pmax was the predictor that accounted for 40.6% (*ρ* = 0.00634) (adjusted R^2^) of the performance variance in the lat pull-down exercise (Table 2).

Likewise, for the bench press exercise, the stepwise multiple regression showed that normF@Pmax and V@Pmax parameters were the predictors that explained 42.3% (*p* = 0.00517) and 65.8% (*p* = 0.00838) of the model variance (adjusted R^2^) in the 100 m mean swimming velocity (dependent variable), respectively (Table 2).

## 4. Discussion

**Figure 1:**
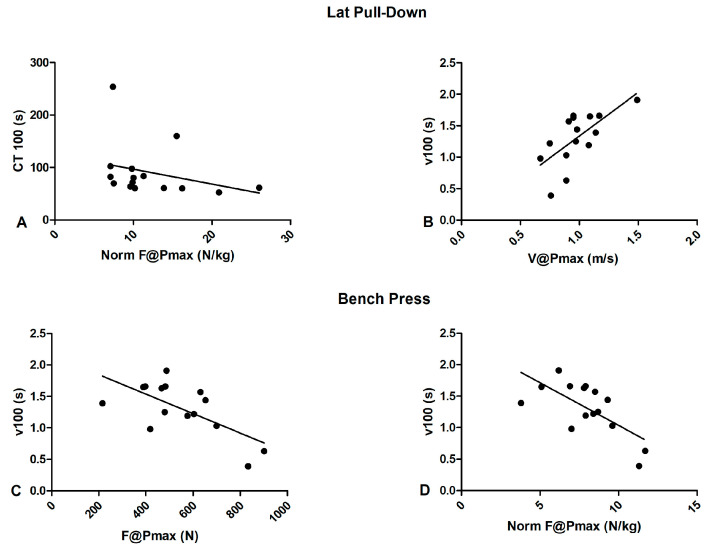
Scatterplot showing the relationships between dryland and swimming outcomes; (**A**) lat pull-down normalized force at maximum power vs. 100 m freestyle chronometric time; (**B**) lat pull-down velocity at maximum power exertion vs. 100 m freestyle mean velocity; (**C**) bench press force at maximum power vs. 100 m freestyle mean velocity; (**D**) bench press normalized force at maximum power vs. 100 m freestyle mean velocity.

The aim of this study was to detect the associations between dryland exercise strength and power outcomes and swimming performance in elite Paralympic swimmers. The main findings revealed a significant relationship in the lat pull-down exercise between the force corresponding to maximum power (F@Pmax) and chronometric time with 100 m freestyle swimming performance (Figure 1)with a significant very large association between 100 m freestyle mean swimming velocity and the V@Pmax in the lat pull-down and the normF@Pmax in the bench press.

Noteworthy, the most informative variables for predicting the 100 m freestyle mean swimming velocity were determined by the lat pull-down V@Pmax (40.6%) and by the bench press V@Pmax and F@Pmax normalized by body weight (65.8% and 42.3%).

Muscular power and force expressed during two common dryland exercises, i.e., the lat pull-down and the bench press, have been shown to correlate with swimming performance in able-bodied swimmers [19,22,32].

Previous studies on able-bodied swimmers showed that the lat pull-down exercise was one of the dryland exercises most related to swimming performance. In fact, the mean propulsive power in this exercise presented a higher significant correlation with swimming velocity (*ρ* = 0.68, *p* = 0.03), with tethered arms-only swimming (*ρ* = 0.69, *p* = 0.03), and tethered whole-body swimming (*ρ* = 0.65, *p* = 0.04) [30]. Following the same line of evidence, Crowe et al. also found that the maximum force expressed during the lat pull-down was positively related (r = 0.643, *p* < 0.05) to swimming performance in 65 division III collegiate swimmers [27]. Lastly, the pull-up exercise, which is similar to the lat pull-down, also presented significant associations in absolute power (r = −0.76), relative power (r = −0.80), relative force (r = −0.77), and mean velocity until muscular failure (r = −0.88) compared to 50 m freestyle swimming time [32]. This association may be explained by the muscles involved in the lat pull-down and freestyle stroke. It is worth noting that for both able-bodied and Paralympic athletes, this on-land exercise (i.e., lat pull-down) involves the activation of the latissimus dorsi muscle, which is an important muscle in swimming related to swimming propulsion and upper body strength during the freestyle stroke [42,43].

In addition, our results provided information that the velocity corresponding to maximum power was the most predictive variable able to explain 40.6% of the performance variance in 100 m freestyle performance, emphasizing the contribution of this on-land exercise to be a good predictor of swimming performance also in Para swimming.

Concerning the bench press, there is a great deal of research supporting the relationship between this exercise and swimming outcomes. For instance, Garrido and colleagues showed that the maximum strength expressed in this exercise (i.e., one maximum repetition) was strictly associated with 25 m (*ρ* = −0.575) and 50 m (*ρ* = −0.586) freestyle sprint time in young competitive swimmers [28]. A significant, moderate relationship (r = 0.538) was found between bench press power and swimming power performance [44]. Moreover, there was a positive association between mean force production in tethered swimming using arms only (*ρ* = 0.73. *p* = 0.02) and whole body (*ρ* = 0.65. *p* = 0.04) [30].

By examining multiple regression analysis in the bench press, it has been shown that mean swimming velocity can be predicted by the normF@Pmax and V@Pmax (42.3% and 65.8%, respectively). Notably, this exercise is important to optimize pectoral major shoulder and triceps muscle strength, which has an influence on the early and late pull phase in freestyle technique [43].

As a matter of fact, upper body muscular strength and power are important features for Paralympic swimmers. The nature of physical impairment may affect muscular force production and functional asymmetries [10]. A severely impaired para-swimmer who is stronger and more powerful can benefit from being physically fit to ameliorate the swimming technique in terms of propulsion, velocity, start, and flip turn [11,16]. A dryland training program focused on developing neuromuscular characteristics is beneficial for elite swimmers to maximize performances over time [13,14,15,17].

Overall, this study may offer novel findings within Paralympic sports science by helping coaches optimize their athletes’ training plans and classifiers to make the athletes’ classification process even more objective. Knowing athletes’ strength and power levels in detail during a dryland context could improve para-swimming classification accuracy.

However, it is worth mentioning that all that glitters is not gold: in both Paralympic and Olympic scenarios, the association between dryland variables and swimming performance should be taken with caution. The reasons why compelling evidence may have found a weak relationship between dryland strength and swimming outcomes might be explained by a lack of specificity that would limit the transfer process between on-land biomechanics to the aquatic context [6,27]. Nevertheless, the consistency between the lat pull-down F@Pmax and V@Pmax or the bench normF@Pmax with swimming chronometric time or mean swimming velocity is encouraging. Additionally, the present investigation shows that lat pull-down V@Pmax, bench press V@Pmax, and normF@Pmax are relevant determinants of swimming velocity in 100 m freestyle performance. It is worth mentioning that the reader should take care in the interpretation of results due to discrepancies between dryland outcomes and swimming performance over time. Future studies might strengthen this association by bringing the time gap between these two measurements even closer.

This research does present some methodological limitations that warrant definitive conclusions. First, the relatively small statistical power due to our participants’ characteristics (elite trained athletes), as well as the absence of mid-to-long races (e.g., 400–800 m) or diverse swimming strokes, suggests further studies to investigate the same outcomes with larger samples in a more variable context [45]. Second, the over-time delay between dryland and in-water measures limits data interpretation. Third, the lack of physiological and biomechanical assessments during swimming performance (e.g., heart rate, lactate blood sample) or an extension of dryland exercises to other body segments (e.g., lower limbs, trunk) reduces the possibility to generalize our results to a more extensive athlete population [46]. Last but not least, the lack of comparison between swimmers with different competitive levels requires caution when considering the present findings, because the environment in which a motor skill is expressed is crucial to developing adaptive strategies for adequately regulating perception and action [47].

## 5. Conclusions

In conclusion, the main findings of the present study indicate a significant association between upper body muscular strength and power outcomes (F@Pmax, normF@Pmax, V@Pmax) in lat pull-down and bench press exercises with swimming performance (swimming chronometric time and mean swimming velocity) in the 100 m freestyle in elite Paralympic swimmers. Furthermore, these dryland variables seem to be also relevant determinants to predict the performance in 100 m freestyle, but the over-time discrepancy between on-land and in-water measurements limits data interpretation. Beyond our results, the relationship between on-land outcomes and aquatic performance should be taken with caution because of the limited transfer process within different environments. Nonetheless, the present research may offer novel findings within Paralympic sports science by helping coaches optimize their athletes’ training plans and classifiers to make the athletes’ classification process more accurate for better supporting Paralympic athletes.

## Figures and Tables

**Table 1:** Spearman correlation coefficients (*p*) between dryland and in-water test outcomes.

Lat Pull-Down	Pmax (W)	NormPmax (W/kg)	F@Pmax (N)	NormF@Pmax (N/kg)	V@Pmax (m/s)
Swimming chronometric time (s)	*ρ* = −0.32	*ρ* = −0.39	*ρ* = −0.49	*ρ* = −0.56 *	*ρ* = −0.41
Mean swimming velocity (m/s)	*ρ* = 0.08	*ρ* = 0.22	*ρ* = 0.11	*ρ* = 0.27	*ρ* = 0.71 **
**Bench Press**					
Swimming chron-ometric time (s)	*ρ* = 0.33	*ρ* = 0.44	*ρ* = 0.18	*ρ* = 0.46	*ρ* = 0.11
Mean swimming velocity (m/s)	*ρ* = −0.44	*ρ* = −0.35	*ρ* = −0.51 *	*ρ* = −0.62 *	*ρ* = 0.28

* = *p* value < 0.05, ** = *p* value < 0.01, Pmax = maximum power, normPmax = normalized maximum power, F@Pmax = force at maximum power, normF@Pmax = normalized force at maximum power, V@Pmax = velocity in maximum power.

**Table 2:** Predictive models for mean swimming velocity (v100) output during lat pull-down and bench press exercises.

**Lat Pull-Down** **(Predictors)**	**R**	**R Square**	**Adjusted R Square**	**Std. Error of the Estimate**	**R Square Change**	**F Change**	**df1**	**df2**	**Sig. F Change**
V@Pmax	0.669	0.448	0.406	0.32160	0.448	10.552	1	13	0.00634
**Bench Press** **(Predictors)**	**R**	**R Square**	**Adjusted R Square**	**Std. Error of the Estimate**	**R Square Change**	**F Change**	**df1**	**df2**	**Sig. F Change**
NormF@Pmax	0.681	0.464	0.423	0.31693	0.464	11.252	1	13	0.00517
V@Pmax	0.841	0.707	0.658	0.24408	0.243	9.919	1	13	0.00838

Note: normF@Pmax = normalized force at maximum power, V@Pmax = velocity in maximum power.

## Data Availability

The data are not publicly available due to privacy reasons.
